# Probing the origin and stability of bivalency in copper based porous coordination network and its application for H_2_S gas capture

**DOI:** 10.1038/s41598-022-19808-y

**Published:** 2022-09-13

**Authors:** Nishesh Kumar Gupta, Eun Ji Kim, Jiyeol Bae, Kwang Soo Kim

**Affiliations:** 1 Department of Environmental Research, University of Science and Technology (UST), Daejeon, 34113 Korea; 2 Department of Environmental Research, Korea Institute of Civil Engineering and Building Technology (KICT), Goyang, 10223 Korea

**Keywords:** Coordination chemistry, Environmental chemistry

## Abstract

A bivalent Cu(I,II) metal–organic framework (MOF) based on the 4,4′,4″-s-Triazine-2,4,6-triyl-tribenzoate linker was synthesized via a solvothermal method. The MOF possessed 43.8% of the Cu sites as Cu^+^ with a surface area of 1257 m^2^ g^−1^. The detailed spectroscopic analysis confirmed dimethylformamide (DMF) solvent as the reductant responsible for Cu^+^ sites in the synthesized MOF. The Cu^+^ sites were easily accessible and prone to oxidation in hot water or acidic gas environment. The MOF showed water-induced structural change, which could be partially recovered after soaking in DMF solvent. The synthesized MOF showed a high hydrogen sulfide (H_2_S) uptake capacity of 4.3 mmol g^–1^ at 298 K and an extremely low H_2_S pressure of 0.0005 bar. The adsorption capacity was the highest among Cu-based MOFs with PCN-6-M being regenerable, which made it useful for deep desulfurization applications. The adsorbed H_2_S was mineralized to sulfide, sulfur, and sulfates, mediated by the Cu^+^/Cu^2+^ redox cycle in the presence of adsorbed water and molecular oxygen. Thus, the study confirmed that DMF as a reductant is responsible for the origin of bivalency in PCN-6-M and possibly in other Cu-based MOFs reported in the literature. Also, the developed MOF could be a potential candidate for flue gas desulfurization and gas purification applications.

## Introduction

The rising concentration of toxic gases in the atmosphere, due to numerous anthropogenic activities, is responsible for deteriorating air quality. Toxic hydrogen sulfide (H_2_S) is a pollutant of immediate concern. H_2_S is a foul odour gas, released from several industrial activities like coal gasification plants, crude oil refineries, livestock farms, food processing units, and municipal sewage treatment facilities. Acute exposure to a low concentration of H_2_S (~ 500 ppm) could cause paralysis and death^1^. H_2_S, upon oxidation, forms oxides of sulfur, which react with fog or cloud droplets to form sulfurous acid (H_2_SO_3_), which in the form of acid rain, causes acidification of soil and water reservoirs^2^. Therefore, the removal of H_2_S has been prioritized to improve air quality and, at the same time, reduce catastrophic events like acid rain.

In the last many years, researchers have studied metal–organic framework (MOF) as an alternative to conventional porous materials like zeolite, silica, and activated carbon for the capture of acidic gases^3^. MOF has found numerous applications in the domain of energy and environment with a prime focus on catalysis^4^, gas separation^5^, and gas capture^6^. MOFs possess the ability to be fine-tuned with specific functionalities, which is highly desirable for their promising application in the selective capture and storage of toxic gaseous pollutants. The flexibility in choosing the metal centre and the organic linker has motivated us to explore a less-known MOF, i.e., PCN-6 (PCN: Porous Coordination Network). PCN-6 is an interwoven MOF constructed through the interaction of 4,4′,4″-s-Triazine-2,4,6-triyl-tribenzoate with Cu(II) ions, leading to the formation of well-known paddlewheel di-copper structural building unit (similar to HKUST-1). These Cu sites are considered open metal sites upon axial ligand removal, which could serve as the binding platform for incoming gas molecules. One of the earliest reported uses of PCN-6 was in hydrogen storage, where the MOF exhibited one of the highest H_2_ storage capacities among MOF materials^7,8^. PCN-6 has been studied for CO_2_ capture as well, showing an uptake capacity of 4.3 mmol g^−1^ at 298 K and 1 bar^9^. Besides gas adsorption, owing to the presence of uncoordinated Cu sites, the non-catenated PCN-6’ form has found application in catalysis^10^. So far, PCN-6 has not been studied for the adsorptive capture of toxic acidic gases like H_2_S, which is the focus of this work.

In recent years, it has been established that Cu-based HKUST-1 has Cu^+^ sites due to the reductive effect of the missing linkers^11^. The presence of Cu^+^ has a significant effect on its gas adsorption/separation behaviour and catalytic activity. Nijem et al. reported preferential adsorption of NO molecules over the Cu^+^ sites compared to Cu^2+^ sites in the presence of water molecules^12^. Daturi et al.studied the redox behaviour of Cu^+^/Cu^2+^ sites in HKUST-1 upon dissociative adsorption of gas molecules^13^. Even in our previously reported studies on Cu-based MOFs, we have witnessed a high proportion of Cu^+^ sites in these MOFs^14–17^. These Cu^+^ sites were present even after thermal treatment at 453 K in air^17^. The notable studies in the literature have suggested that Cu^+^ sites could form during thermal activation, exposure to X-ray irradiation, and reductive gas environments. In our case, we have reported a high proportion of Cu^+^ sites even in the absence of these conditions. Previously. we suggested that the creation of Cu^+^ sites was probably due to the reductive effect of dimethylformamide (DMF) molecules or the low surface area of the MOF, which restricted the entry of water molecules^15,16^. We have witnessed a large proportion of Cu^+^ sites in the PCN-6 MOF, which is important to explore considering similarities with the HKUST-1 structure. Here, we have decided to explore the reason for Cu^+^ sites in PCN-6 and their stability in different oxidative environments.

We have fabricated a Cu(I,II)-PCN-6 (PCN-6-M) with mixed Cu oxidation states using a facile solvothermal reaction. The MOF was well-characterized using various microscopic and spectroscopic techniques to deduce its physicochemical properties. Moreover, the study has investigated the reason behind the presence of Cu(I) sites in the MOF and their stability upon exposure to hot water and SO_2_ gas. The synthesized PCN-6-M was studied for the adsorptive removal of H_2_S gas in breakthrough studies at 298 K for deep desulfurization applications. Besides uptake behaviour, the study is focused on deducing the adsorption mechanism and MOF stability after gas exposure. This work has shown the potential of PCN-6-M in air decontamination applications.

## Methods

### Chemicals

Copper(II) nitrate trihydrate (Cu(NO_3_)_2_·3H_2_O), *N*,*N*-dimethyl formamide (DMF), and chloroform (CH_2_Cl_2_) were purchased from Samchun Pure Chemicals, Korea. 4,4′,4″-s-Triazine-2,4,6-triyl-tribenzoic acid (H_3_TATB, purity ~ 95%) was procured from Sigma Aldrich, Germany. H_2_S (0.05 vol.%) and SO_2_ gas (0.01 vol.%) in nitrogen were purchased from Union gas, Korea. All the chemicals were of analytical grade and used without any further purification.

### Synthesis of MOF

Exactly 2.58 g of Cu(NO_3_)_2_·3H_2_O and 0.50 g of H_3_TATB were dissolved in 300 mL of DMF solution. The solution was placed in a 1 L Teflon-lined autoclave and heated at 348 K for 6 h. After cooling to room temperature, the blue crystals were isolated by decanting the mother liquor and washing with DMF (2 × 30 mL). The solid product was immersed in 10 mL of CH_2_Cl_2_ for 2 days, during which CH_2_Cl_2_ was replaced twice. After solvent removal, the sample was dried at 343 K for 24 h. The MOF was stored at 363 K in a glass vial.

### Analytical instruments

The surface morphology of MOF was deduced by field emission scanning electron microscopy (FE-SEM) (Hitachi S-4300, Hitachi, Japan) after coating the sample with a gold-platinum alloy by ion-sputtering (E-1048 Hitachi ion sputter). The 2D elemental mapping was done by energy-dispersive X-ray spectroscopy (EDS) (X-Maxn 80T, Oxford Instruments, United Kingdom). Field emission transmission electron microscope (FE-TEM) micrograph was collected over FE-TEM (JEM-2010F, JEOL Ltd., Japan). The diffraction patterns were obtained on an Ultima IV X-ray diffractometer (Rigaku, Japan) with Cu-Kα_1_ radiation (λ = 1.5406 Å). For Brunauer–Emmett–Teller (BET) analysis, N_2_ adsorption–desorption isotherm was measured at 77 K in a Gemini 2360 series instrument (Micromeritics, United States) after degassing at 473 K for 8 h. Fourier-transform infrared (FTIR) spectra were recorded over a Cary670 FTIR spectrometer (Agilent Technologies, United States) using KBr pellets. X-ray photoelectron spectroscopy (XPS) analysis was performed on a K-alpha XPS instrument (Thermo Fisher Scientific, United Kingdom) with a monochromatic Al Kα X-ray source. The pressure was fixed to 4.8 × 10^−9^ mbar. Spectra were charge corrected to the main line of the C 1s spectrum (aromatic carbon) set to 284.7 eV. Spectra were analysed using CasaXPS software (version 2.3.14). GL(*p*) = Gaussian/Lorentzian product formula where the mixing is determined by *m* = *p*/100, GL(100) is a pure Lorentzian while GL(0) is pure Gaussian. We have used GL(30).

### Experimental protocols

H_2_S gas uptake measurement was performed at 298 K by placing a known mass of MOF between glass wools in a Pyrex tube. H_2_S gas (0.05 vol.%) was passed through it at a fixed flow rate and the outgoing gas was analysed by an H_2_S gas analyser (GSR-310, Sensoronic, Korea) with the detection limit of 0.1 ppm. The H_2_S adsorption capacity was calculated using the following equation^14^.

1 $$q = \frac{{C_{0} Q}}{m}\mathop \smallint \limits_{0}^{{t_{b} }} \left( {1 - \frac{{C_{t} }}{{C_{0} }}} \right)dt$$

where *C*_0_—initial concentration (mg L^−1^), *C*—concentration at time ‘*t*’ (mg L^−1^), *Q*—flowrate (L min^−1^), *m*—the mass of adsorbent (g), and *t*_b_—breakthrough time (s).

The SO_2_-exposed sample was prepared by passing SO_2_ gas (0.01 vol%) at a flow rate of 0.1 L min^−1^ for 4 h.

## Results and discussion

**Figure 1:**
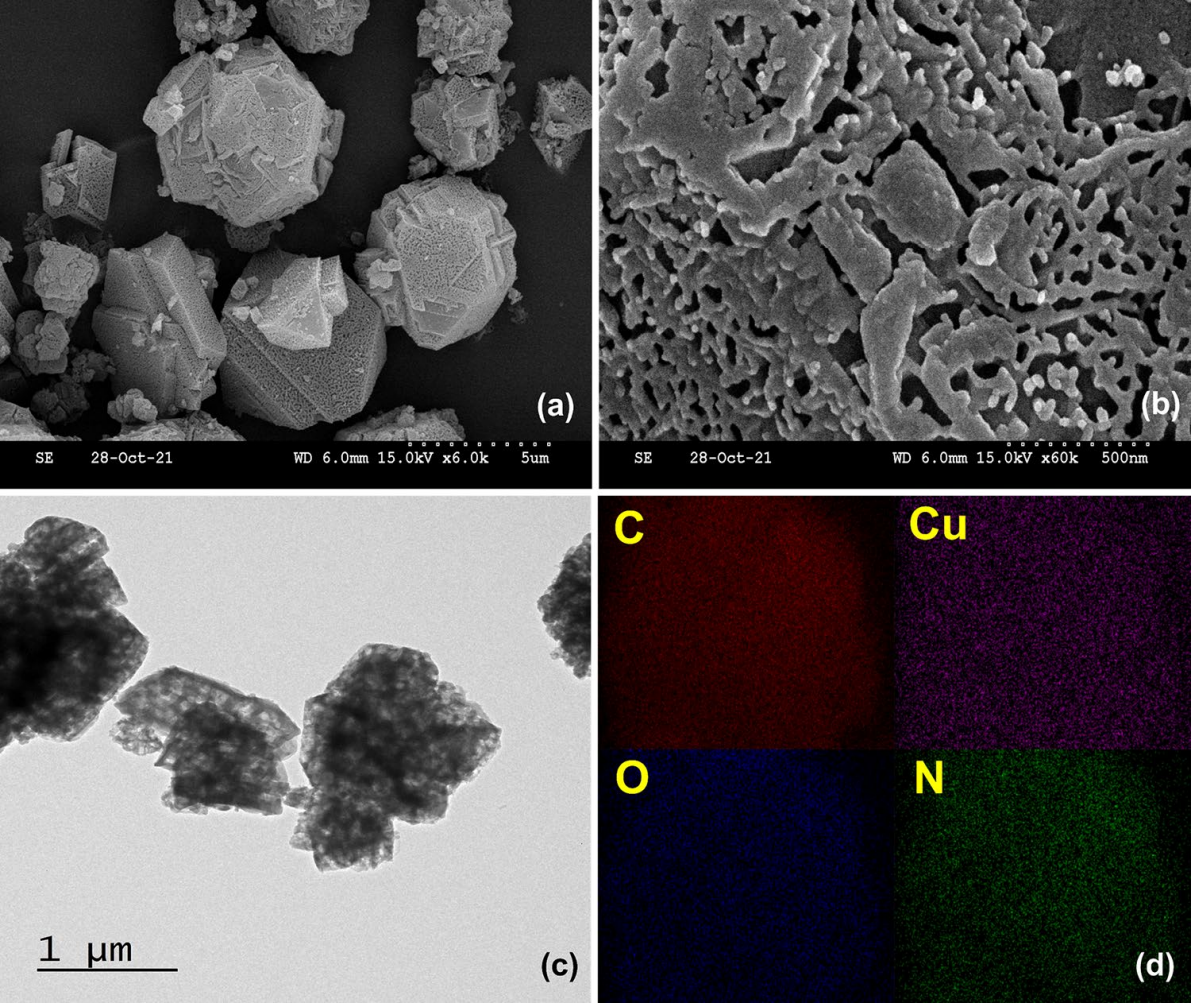
(**a, b**) SEM micrographs; (**c**) TEM micrograph; (**d**) SEM-EDAX analysis of PCN-6-M.

The SEM micrograph of PCN-6-M at low resolution showed micron-size particles in the form of multi-facet balls with a flat and smooth surface (Fig. 1a). The previous study on PCN-6-M formation reported microparticles with irregular morphology^9,10^. A closer inspection of the MOF surface at high resolution showed an irregular net-like structure on the surface (Fig. 1b). This net-like porous structure was further confirmed by the TEM micrograph (Fig. 1c). The 2D elemental mapping showed a uniform presence of C, O, Cu, and N in the MOF (Fig. 1d).

**Figure 2:**
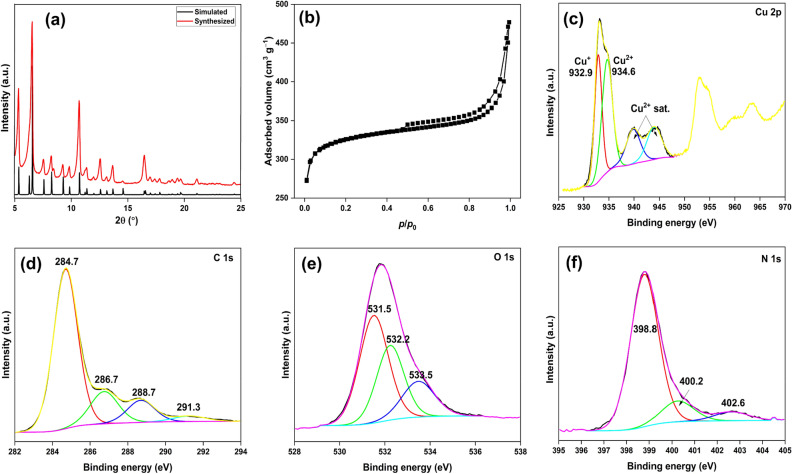
(**a**) PXRD pattern; (**b**) N_2_ adsorption–desorption isotherm; high-resolution XPS (**c**) Cu 2p spectrum; (**d**) C 1s spectrum; (**e**) O 1s spectrum; (**f**) N 1s spectrum of PCN-6-M.

The pattern of synthesized MOF fully matched with the simulated pattern, confirming the formation of highly pure PCN-6-M (Fig. 2a)^8^. The N_2_ adsorption–desorption isotherm of PCN-6-M indicated Type IVa sorption behaviour as opposed to Type I reported for PCN-6 (Fig. 2b)^9,18^. Thus, as opposed to the observed microporosity in the previously reported PCN-6, the MOF fabricated in this study has mesoporosity (average pore diameter ~ 11.3 Å)^19^. For this reason, the BET surface area (1257 m^2^ g^−1^) and pore volume (0.709 cm^3^ g^−1^) of PCN-6-M were much lower than that of PCN-6^9^. The high-resolution XPS (HRXPS) Cu 2p spectrum has Cu 2p_3/2_ peak deconvoluted into two contributions at 932.9 and 934.6 eV for Cu^+^ (43.8%) and Cu^2+^ (56.2%) ions, respectively. The shake-up satellite peaks (*p* → *d* hybridization of *d*^9^ configuration) observed in the range of 935–945 eV were due to the Cu^2+^ ions (Fig. 2c, Table S1)^17^. The HRXPS C 1s spectrum has four peaks at 284.7, 286.7, 288.7, and 291.3 eV for C=C (69.6%), C–O/C–N (16.2%), –O–C=O (10.9%), and π–π^*^ (3.2%), respectively, from the TATB^3−^ linker (Fig. 2d)^20,21^. The HRXPS O 1*s* spectrum has three contributions at 531.5, 532.2, and 533.5 eV for Cu–O (49.1%), C–O (32.3%), and H_2_O (18.5%), respectively (Fig. 2e)^17^. The HRXPS N 1s spectrum has two main contributions for C–N=C from the heterocyclic skeleton and protonated C–N^+^H=C species at 398.8 (81.3%) and 400.2 eV (12.8%), respectively (Fig. 2f, Table S2)^22^. A minor contribution at 402.6 eV (5.9%) was probably due to the amide groups of DMF solvent molecules bound to the Cu sites^23^.

**Figure 3:**
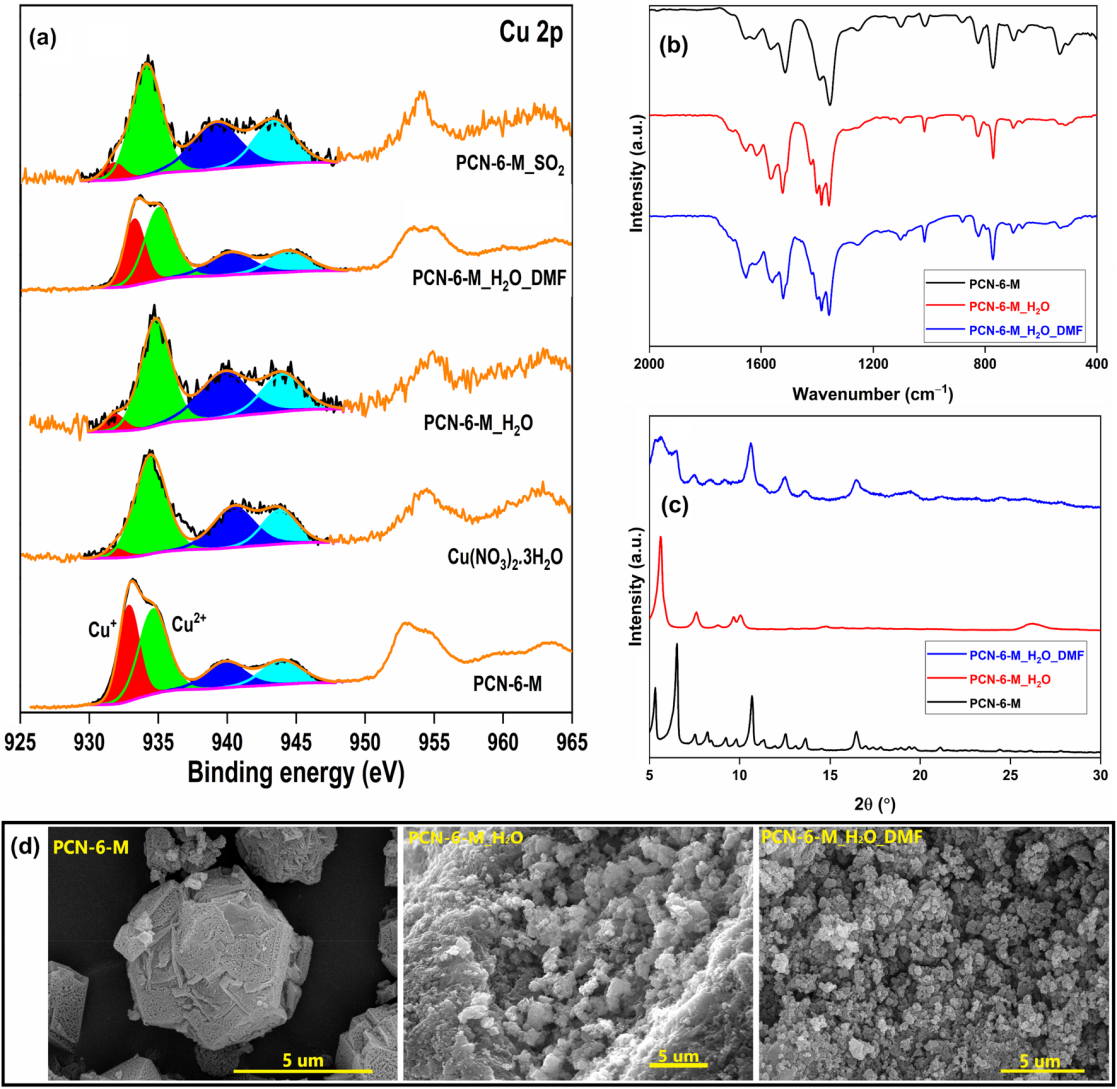
(**a**) HRXPS Cu 2p spectra of PCN-6-M in different environments; (**b**) FTIR spectra; (**c**) PXRD patterns; (**d**) SEM micrographs of PCN-6-M and after soaking in water, followed by soaking in DMF at 343 K.

In the literature, multiple studies have reported the reduction of Cu(II) to Cu(I) in MOFs as a two-step process using reducing agents^24,25^. Here, we have observed a large proportion of Cu as Cu^+^ (43.8%) without any additional reducing agent during the synthesis process. A large proportion of Cu^+^ ions in this MOF was either due to Cu^+^ ions in the salt or the reducing effect of DMF solvent. In our previously published work on the fabrication of Ag–Cu–MOF, a significant proportion of Cu^+^ and Ag^0^ species observed in the MOF was linked to the reducing effect of DMF solvent^16^. Also, El-Yazeed and Ahmed^26^ reported similar observations during the synthesis of Cu-Ag MOF/MCM-41 composites. Though on many occasions, the mixed oxidation state of Cu ions in MOFs is linked to the reducing behaviour of DMF, no efforts have been made to verify this claim. Here, we have tried to confirm the role of DMF as a reductant responsible for the Cu^+^ sites. Firstly, the HRXPS Cu 2p spectrum of Cu nitrate salt was analyzed to confirm its Cu oxidation state (Fig. 3a). The Cu salt has a minor fraction of Cu^+^ (~ 5%) in the sample (Table S1), which could be due to the reduction of Cu^2+^ ions during X-ray exposure^12^. Thus, it was obvious that Cu salt has no role to play towards Cu^+^ sites in the MOF and the reductive effect of DMF could be responsible for it.

To better understand the stability and accessibility of Cu^+^ sites in the MOF, the MOF sample (100 mg) was soaked in 10 mL of hot water (343 K) till the sample dried fully, which was then analyzed. XPS analysis showed a major drop in the Cu^+^ proportion, i.e., from 43.8% in the fresh sample to 9.2% in the soaked sample, because of oxidation in the presence of hot water^27^. The result confirmed that the Cu^+^ sites are fully accessible to small molecules and prone to oxidation. The FTIR analysis showed a drop in the intensity of the 1654 cm^−1^ band with the appearance of a shoulder around 1709 cm^−1^, which could be related to the breaking of the metal-carboxylate bond and formation of free carboxylic groups (Fig. 3b). Similar results are available for HKUST-1 in the presence of moisture^28^. These changes led to significant variations in the crystal structure, which is evident from the PXRD pattern of PCN-6-M_H_2_O (Fig. 3c), which is analogous to the reported water-induced structural changes in HKUST-1^29^. The accessibility of Cu^+^ sites was further confirmed by exposing the MOF sample to SO_2_ gas, where a large drop in the Cu^+^ sites (8.9%) was observed after exposure. The oxidation of Cu^+^ sites in HKUST-1 to Cu^2+^ in acidic gas (NO_2_) environment is well-documented in the literature^13^. Thus, the detailed investigation confirmed that the Cu^+^ sites were unstable and prone to oxidation in moisture or acidic environment.

The role of DMF as a reductant was confirmed by soaking the PCN-6-M_H_2_O sample (100 mg) in DMF (10 mL) at 343 K till dryness (sample labelled as PCN-6-M_H_2_O-DMF). From the Cu 2p spectrum, it was clear that the proportion of Cu^+^ in the MOF significantly improved from 9.2% in PCN-6-M_H_2_O to 38.3% after DMF soaking (Fig. 3a, Table S1). Though the initial Cu^+^ proportion of 43.8% in the fresh sample could not be regained, the achieved proportion was more than enough to validate that the DMF solution does play a strong role in the reduction of Cu^2+^ sites to Cu^+^ in PCN-6-M. The SEM micrograph of PCN-6-M_H_2_O showed significant alterations in the surface morphology of PCN-6 after water soaking, which was irreversible as we were not able to regain the original morphology of PCN-6-M even after DMF soaking (observed in PCN-6-M_H_2_O_DMF) (Fig. 3d). However, this soaking exercise has a low impact on the PXRD pattern as the diffraction peaks related to the fresh PCN-6-M were recovered after the DMF soaking along with the remnant of PCN-6-M_H_2_O peaks (Fig. 3c). Thus, apart from regaining the Cu^+^ sites, the MOF showed the potential of partial recovery of its crystal structure upon DMF soaking. These key spectroscopic findings concluded the role of DMF as a reductant during the Cu-based MOF synthesis. Moreover, the study further confirmed that the crystal structure of fabricated PCN-6-M (changed after soaking in hot water) could be regained effectively by DMF solvent. Such a phenomenon is well-established for HKUST-1, which upon water-induced structural degradation (0.5 h exposure to water at 358 K), could be reconstructed after soaking in ethanol at room temperature^30^.

**Figure 4:**
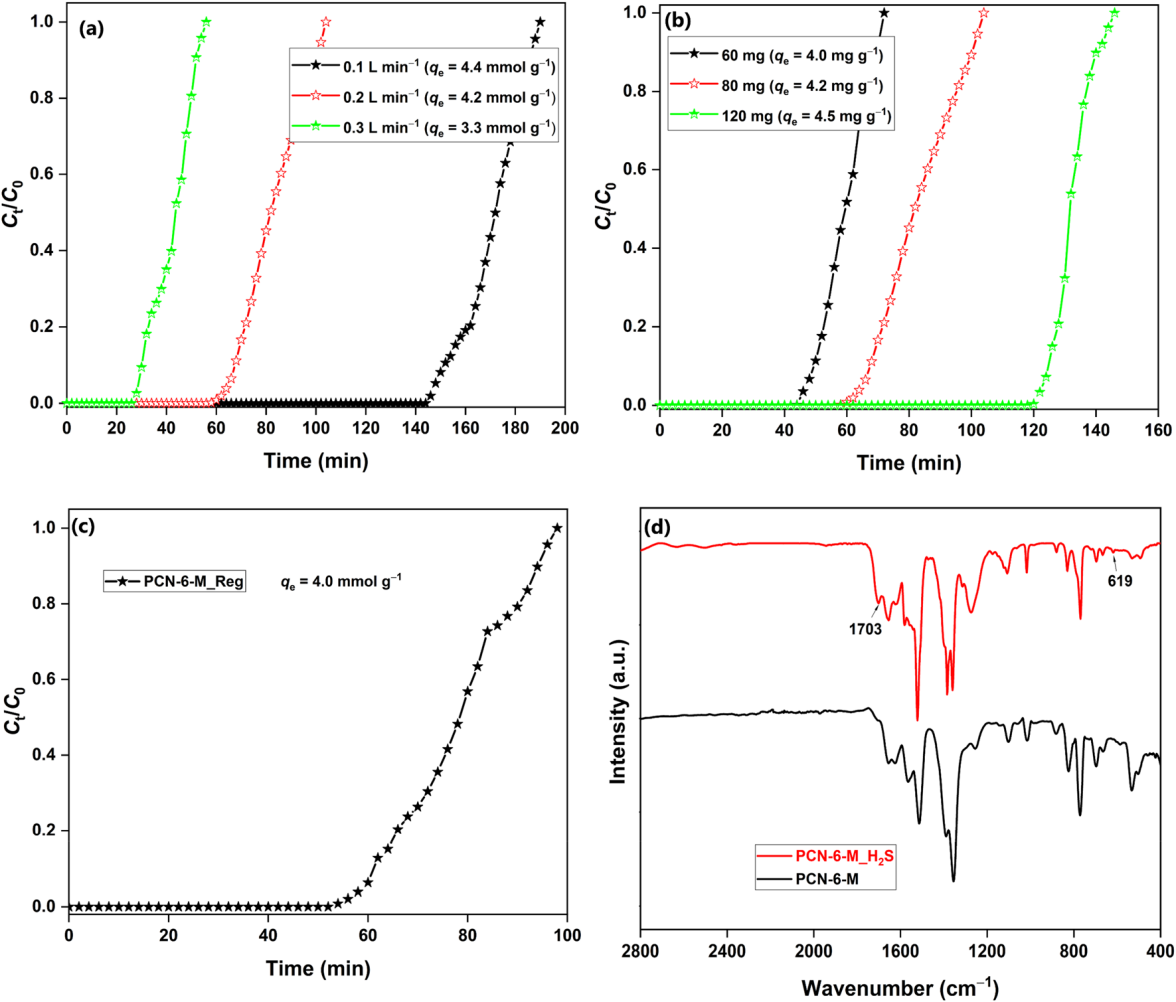
Effect of (**a**) flowrate; (**b**) MOF mass on H_2_S adsorption capacity of PCN-6-M; (**c**) H_2_S breakthrough curve of regenerated PCN-6-M; (**d**) FTIR spectra of fresh and H_2_S-adsorbed PCN-6-M (conditions: 80 mg MOF, 0.2 L min^–1^ flow rate, changed otherwise).

The synthesized PCN-6-M was studied for the capture of H_2_S gas in the breakthrough study at 298 K and extremely low H_2_S pressure of 0.0005 bar (Fig. 4a,b). The effect of flowrate was studied by fixing the MOF mass to 80 mg and varying the flowrate between 0.1 and 0.3 L min^–1^ (Fig. 4a). The adsorption capacity decreased from 4.4 to 3.3 mmol g^–1^ with the increasing flow rate from 0.1 to 0.3 L min^–1^. The negative effect of the increasing flow rate is associated with a drop in the gas retention time, which reduces the adsorbate-adsorbent interaction^20^. The effect of adsorbent loading was studied in the 60–120 mg range with a flowrate of 0.2 L min^–1^ (Fig. 4b). The adsorption capacity improved with the increasing MOF loading. The increasing mass increases the bed length, which favours the adsorption process by enhancing the gas retention time^31^. The MOF has a high breakthrough adsorption capacity of 4.5 mmol g^–1^, which makes the MOF suitable for deep desulfurization applications. The H_2_S uptake capacity of PCN-6-M is the highest among reported Cu-based MOFs like HKUST-1 (3.4 mmol g^–1^)^32^, Cu(BDC)_0.5_(BDC-NH_2_)_0.5_ (3.8 mmol g^–1^)^15^, Cu-BDC(ted)_0.5_ (1.8 mmol g^–1^)^33^, and CuBDC (3.1 mmol g^–1^)^14^. The H_2_S-adsorbed PCN-6-M sample was reconstructed by heating it in a 0.04 mol L^–1^ Cu^2+^ solution in DMF (10 mL) at 348 K by placing it in a capped glass vial for 3 h. The strategy was highly effective as blue crystals of pristine PCN-6-M were recovered after 3 h. The regenerated MOF sample showed an H_2_S uptake capacity of 4.0 mmol g^–1^, which makes the adsorption process highly lucrative for deep desulfurization application (Fig. 4c). In the FTIR spectrum, new bands appeared at 619 and 1703 cm^–1^ for Cu–S^34^ and free –COOH groups, respectively. Thus, the Cu–S bond formation in the MOF was mediated by the breaking of the Cu–carboxylate bond (Fig. 4d).

**Figure 5:**
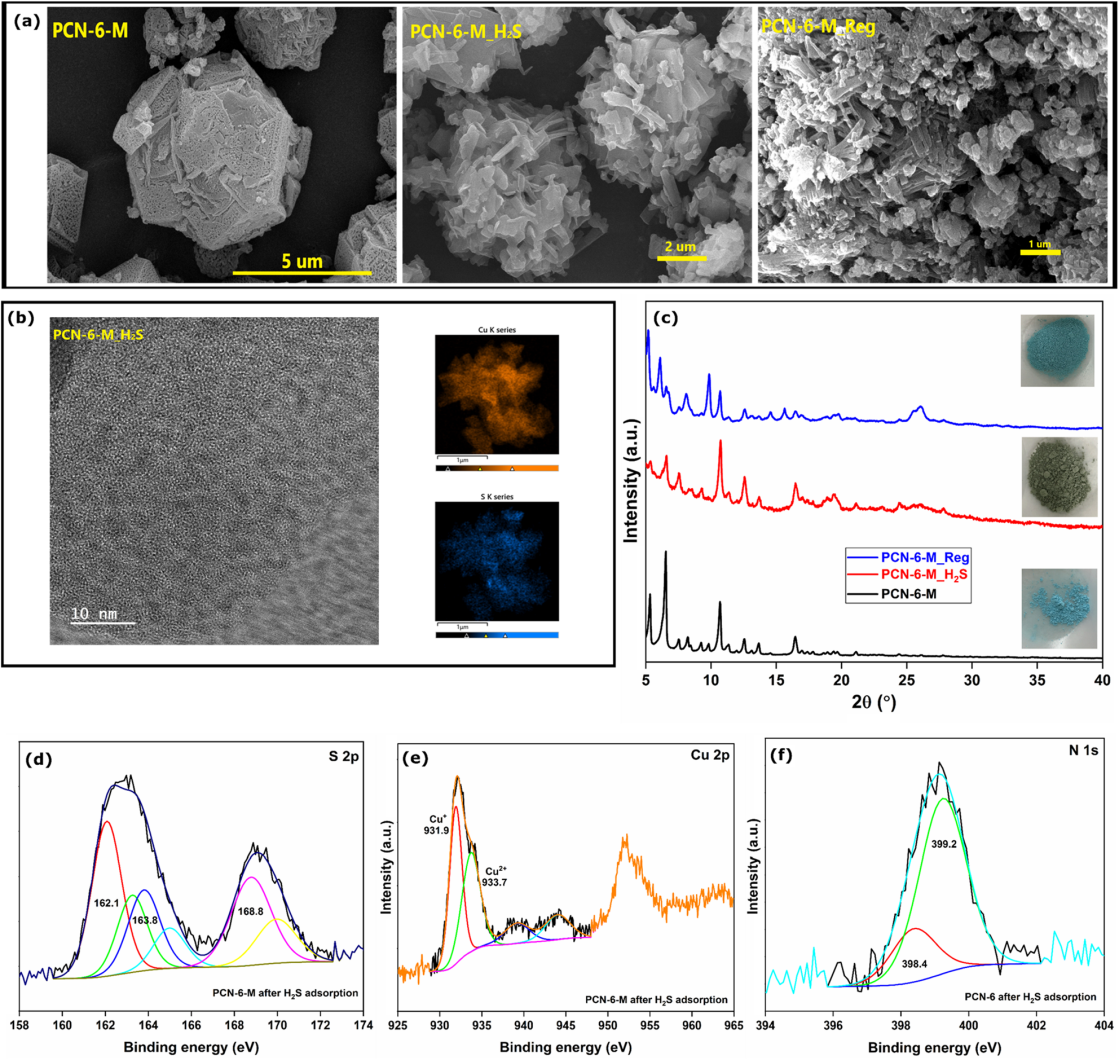
(**a**) SEM micrographs (**b**) High-resolution TEM and TEM-EDAX analysis of PCN-6-M_H_2_S; (**c**) PXRD patterns; (**d**) S 2p; (**e**) Cu 2p; (**f**) N 1s spectra of PCN-6-M after H_2_S adsorption.

The SEM micrographs showed the fracturing of multi-facet balls (PCN-6-M) upon H_2_S exposure (PCN-6-M_H_2_S), which showed that the morphology was severely compromised during H_2_S adsorption. This morphology remained unrecoverable even after the regeneration of the sample, which showed a disordered arrangement of microrods (Fig. 5a). Thus, it was confirmed that once the morphology of PCN-6-M is lost then it is impossible to regain under any condition studied here. The HRTEM image of PCN-6-M_H_2_S confirmed the absence of CuS/Cu_2_S nanoparticles, which was following the PXRD result. The EDAX mapping confirmed the uniform distribution of the ‘S’ element over the ‘Cu’ map (Fig. 5b). The PXRD pattern of H_2_S-adsorbed MOF showed an intensity loss for the low-angle diffraction peaks with the absence of CuS/Cu_2_S, suggesting a partial framework collapse after H_2_S adsorption^35^. Also, the MOF colour changed from ocean blue to dark green upon H_2_S adsorption. Such a colour change has been witnessed for MOF-199 as well^17^. Upon regeneration, we observed regain in the intensity of low-angle peaks for the PCN-6-M_Reg sample. Though the diffraction pattern did not fully overlap with that of the pristine PCN-6-M, it is important to note that the heating was done for 3 h and in the presence of sulphur byproducts as impurities. However, we could confirm the regeneration of Cu-sites from the formation of ocean blue crystals (Fig. 5c).

The HRXPS S 2p spectrum was deconvoluted into three sets of doublets, which were assigned to the 2p_3/2_ and 2p_1/2_ signals of sulfide, elemental sulfur, and sulfate (Fig. 5d). The S 2p_3/2_ peak (43.0%) at 162.1 eV was attributed to the sulfide (S^2–^), while the 2p_3/2_ peak at 163.8 eV (24.0%) was assigned to the elemental sulfur (S^0^). The S 2p_3/2_ peak at 168.8 eV (33.0%) was attributed to the sulfate species (SO_4_^2–^)^20,36^. The HRXPS Cu 2p spectrum has Cu^+^ and Cu^2+^ peaks at 931.9 and 933.7 eV, respectively, with a redshift of ~ 1.0 eV compared to the fresh sample (Fig. 5e). The large redshift in the peak position confirmed a strong interaction of S^2–^ ions with both the monovalent and divalent Cu-sites^37^. Similar behaviour was reported for H_2_S adsorption over Cu(BDC)_0.5_(BDC-NH_2_)_0.5_ in our previously reported study ^15^. The H_2_S adsorption over the MOF was initiated by the dissociation of H_2_S molecules to sulfide ions and their subsequent binding to the Cu-sites. This dissociation was facilitated by the N-atom of the heterocyclic linker through a proton-extraction mechanism. For this reason, in the HRXPS N 1s spectrum, the proportion of the C–N^+^H=C peak increased to 77.7% as opposed to 12.8% in the fresh sample (Fig. 5f). A large presence of S^0^ in the MOF was due to the oxidation of S^2–^ to S^0^, which was mediated by the reduction of Cu^2+^ to Cu^+^ sites. This redox reaction was inferred from the increased Cu^+^ proportion (51.9 opposed to 43.8% in the fresh sample) in the H_2_S-adsorbed sample. Moreover, the sulfur further oxidized to sulfate in the presence of molecular oxygen and water-mediated by the Cu^2+^/Cu^+^ redox cycle^15^.

## Conclusion

In this study, we have reported a bivalent Cu(I,II)-PCN-6 MOF constructed via a solvothermal reaction between Cu(II) ions and 4,4′,4″-s-Triazine-2,4,6-triyl-tribenzoate linker. The PXRD pattern of PCN-6-M matched well with the reported pattern of PCN-6. The prepared MOF has a surface area of 1257 m^2^ g^−1^ with ~ 44% of the Cu sites in the monovalent state. The spectroscopic analysis confirmed that the Cu^+^ sites in the MOF originated from the reductive effect of DMF solvent during the synthesis process. Moreover, the Cu^+^ sites were readily accessible and prone to oxidation under moist or acidic conditions. The MOF showed a water-induced structural change, which could be partially recovered after soaking in DMF solvent, which was confirmed by PXRD analysis. The MOF possessed a high H_2_S adsorption capacity of 4.3 mmol g^–1^ at 298 K and 0.0005 bar, which was the highest among Cu-based MOFs in similar experimental conditions. The XPS analysis confirmed the formation of sulfide, sulfur, and sulfate as the H_2_S adsorption-oxidation species, mediated by the Cu^+^/Cu^2+^ redox cycle in the presence of pre-adsorbed oxygen and water. The study highlighted the role of DMF as a reductant during the Cu-based MOF synthesis and at the same time reported a regenerable MOF as a potential candidate for deep desulfurization applications.

## Supplementary Information

Supplementary Information.
