# Supplementary material for: Probing the origin and stability of bivalency in copper based porous coordination network and its application for H2S gas capture
Source: Sci Rep. 2022 Sep 13;12:15388. doi: 10.1038/s41598-022-19808-y (PMC9470748; doi:10.1038/s41598-022-19808-y)
Supplement: Supplementary file 1 — Supplementary Information. [file 41598_2022_19808_MOESM1_ESM.docx]

**Probing the Origin and Stability of Bivalency in Copper Based Porous Coordination Network and its Application for H_2_S Gas Capture**

Nishesh Kumar Gupta^1,2^, Eun Ji Kim^1,2^, Jiyeol Bae^1,2*^, Kwang Soo Kim,^1,2**^

^1^Department of Environmental Research, University of Science and Technology (UST), Daejeon 34113, Korea

^2^Department of Environmental Research, Korea Institute of Civil Engineering and Building Technology (KICT), Goyang 10223, Korea

**Table S1.** HRXPS Cu 2p assignments for PCN-6-M samples.

| **Samples** | **Assignment** | **E_B_ (eV)** | **FWHM (eV)** | **At. %** |
| --- | --- | --- | --- | --- |
| **PCN-6-M** | **Cu2p_3/2_** _Cu+_ | 932.9 | 1.7 | 43.8 |
|  | **Cu2p_3/2_** _Cu2+_ | 934.6 | 2.5 | 56.2 |
|  | **Cu2p_3/2_** _Satellite Cu2+_ | 939.8 | 3.3 | - |
|  | **Cu2p_3/2_** _Satellite Cu2+_ | 944.0 | 3.4 | - |
| **Cu(NO_3_)_2_.3H_2_O** | **Cu2p_3/2_** _Cu+_ | 931.9 | 1.6 | 5.0 |
|  | **Cu2p_3/2_** _Cu2+_ | 934.4 | 2.6 | 95.0 |
|  | **Cu2p_3/2_** _Satellite Cu2+_ | 940.4 | 3.5 | - |
|  | **Cu2p_3/2_** _Satellite Cu2+_ | 943.8 | 3.0 | - |
| **PCN-6-M_H_2_O** | **Cu2p_3/2_** _Cu+_ | 931.8 | 1.6 | 9.2 |
|  | **Cu2p_3/2_** _Cu2+_ | 934.8 | 2.5 | 90.8 |
|  | **Cu2p_3/2_** _Satellite Cu2+_ | 939.8 | 4.0 | - |
|  | **Cu2p_3/2_** _Satellite Cu2+_ | 944.0 | 3.5 | - |
| **PCN-6-M_H_2_S** | **Cu2p_3/2_** _Cu+_ | 931.9 | 1.8 | 51.9 |
|  | **Cu2p_3/2_** _Cu2+_ | 933.7 | 2.6 | 48.1 |
|  | **Cu2p_3/2_** _Satellite Cu2+_ | 939.2 | 3.5 | - |
|  | **Cu2p_3/2_** _Satellite Cu2+_ | 944.0 | 3.5 | - |
| **PCN-6-M_SO_2_** | **Cu2p_3/2_** _Cu+_ | 931.8 | 1.6 | 8.9 |
|  | **Cu2p_3/2_** _Cu2+_ | 934.1 | 2.5 | 91.1 |
|  | **Cu2p_3/2_** _Satellite Cu2+_ | 939.1 | 4.0 | - |
|  | **Cu2p_3/2_** _Satellite Cu2+_ | 943.4 | 3.5 | - |
| **PCN-6-M_H_2_O_DMF** | **Cu2p_3/2_** _Cu+_ | 933.2 | 1.7 | 38.3 |
|  | **Cu2p_3/2_** _Cu2+_ | 935.0 | 2.5 | 61.7 |
|  | **Cu2p_3/2_** _Satellite Cu2+_ | 940.2 | 3.6 | - |
|  | **Cu2p_3/2_** _Satellite Cu2+_ | 944.4 | 3.4 | - |

**Table S2.** HRXPS N 1s assignments for PCN-6-M samples.

| **Samples** | **Assignment** | **E_B_ (eV)** | **FWHM (eV)** | **At. %** |
| --- | --- | --- | --- | --- |
| **PCN-6-M** | C−N=C | 398.8 | 1.4 | 81.3 |
|  | C−N^+^H=C | 400.2 | 1.6 | 12.8 |
|  | Amide of DMF | 402.6 | 1.8 | 5.9 |
| **PCN-6-M_H_2_S** | C−N=C | 398.4 | 1.5 | 22.3 |
|  | C−N^+^H=C | 399.2 | 1.6 | 77.7 |
| **PCN-6-M_H_2_O** | C−N=C | 398.4 | 1.5 | 77.8 |
|  | C−N^+^H=C | 399.2 | 1.6 | 22.2 |
